# Aggressiveness in Adopted and Non-Adopted Teens: The Role of Parenting, Attachment Security, and Gender

**DOI:** 10.3390/ijerph18042034

**Published:** 2021-02-19

**Authors:** Miriam Gallarin, Barbara Torres-Gomez, Itziar Alonso-Arbiol

**Affiliations:** Faculty of Psychology, University of the Basque Country UPV/EHU, 20018 Donostia-San Sebastián, Spain; barbara.torres@ehu.eus (B.T.-G.); itziar.alonso@ehu.eus (I.A.-A.)

**Keywords:** aggressiveness, attachment, parenting practices, adoption, early adolescence, gender

## Abstract

The aim of this study was to examine the relationship among aggressiveness, parenting practices, and attachment security in adolescents, assessing maternal and paternal effects separately. Two different subsamples of adolescents between 12 and 16 years old participated in the study (*n* = 157): 67 adopted adolescents (61.2% girls) and 90 non-adopted adolescents (56.7% girls). Partial and full mediation models were analyzed in multi-group structural equation models (using maximum likelihood estimates), allocating non-adoptive and adoptive adolescents into two different groups. Results showed that whereas acceptance/involvement of each parent predicted attachment security towards the corresponding parental figure, only the father’s coercion/imposition predicted aggressiveness, and only attachment security to the mother was a (negative) predictor of adolescent’s aggressiveness. The partial mediation model provided the most parsimonious explanation for the data, showing no differences between adopted and non-adopted subsamples and supporting a good model fit for both boys and girls in a multi-group invariance analysis. The implications of these results are discussed in light of the protective effects of care relationships in early adolescence (vs. late adolescence) as well as the differential role of parent figures.

## 1. Introduction

Aggressiveness is a complex, polymorphic and multidimensional phenomenon characterized by individual variability in the intensity of the manifestations in each of its dimensions: emotional, cognitive, and behavioral [1,2,3]. Numerous studies conclude that inadequate management of aggressiveness is related to the development of several individual and relational difficulties in the short, medium, and long term [4,5,6]. Taking into account that in adolescence, there is an increase in aggressive behaviors [1,7], it is of special interest to identify possible factors that could contribute to such an increase. Actually, it is well established that aggressiveness and aggression phenomena are the products of gene-environment interactions, being the genetic risk for aggression enlarged by an adverse milieu or decreased by a favorable one [8]. In the environment domain, it is known that the quality of family relationships established during childhood and adolescence makes a relevant contribution to the development of aggressiveness [9,10], but different stages of adolescence require adjusted parenting specific to evolving developmental needs [11]. In consonance with such specificity, the effects of parental behaviors on early and late adolescents’ aggressiveness also need to be assessed.

Researchers have addressed the study of attachment and parental practices as the most important family aspects to explain aggressiveness and/or the appearance of associated variables (problem behavior, aggressive behavior, externalizing problems, criminal behavior, delinquency, and violence) in sons and daughters. While attachment refers to the (in)security of the affective bond established with the main caregivers [12], parental practices include the wide repertoire of behaviors that parents carry out in child-rearing [13]. These practices are usually grouped under the labels of acceptance/involvement—also called responsiveness [14] or warmth [15]—and coercion/imposition—or demandingness [16] and strictness [17]. There is ample evidence to support the effect of both attachment and parenting on aggressiveness. For example, in the review carried out by Savage [18], findings suggest a very consistent negative association between attachment security and violent behavior. Regarding parental practices, aggressiveness has been inversely linked to acceptance and involvement behaviors and positively associated with coercion and imposition of care figures [19,20]. Warmth, communication, and autonomy support have recently been identified as the main aspects of positive parenthood [21], and there is a clear association between positive parenthood and lower rates of relational aggression [22]. In this same line, a recent study highlights the positive impact of parental warmth, even with aggressive adolescents [23]. In a meta-analysis carried out by Pinquart [24], aspects related to involvement (e.g., parental warmth) and to imposition (e.g., punitive control) were associated with behavioral problems in general. Finally, and regarding the relationship between attachment and parental practices, strong positive associations between secure attachment and variables related to acceptance and involvement (responsiveness, support for autonomy, and behavioral control); and negative associations with variables related to coercion and imposition (punitive control) were observed in the meta-analysis by Koehn and Kerns [25]. As a consequence of parental practices displayed by each parental figure and of the dyadic relationship established with their son/daughter (e.g., tone of voice and body language), a specific emotional climate or parental style is developed. Parental styles can contribute to the quality of the emotional bond established in the caregiver-child dyad, helping the child in his/her development of an internal model of attachment [26].

For this reason, apart from all direct relationships between attachment and parental practices (and between each variable and aggressiveness), some studies have proposed moderation and mediation models to explain aggressiveness in childhood. For example, Kochanska and colleagues, in samples of infant and early school-aged children (5–80 months), found differential effects of parenting practices on antisocial behaviors due to attachment, attachment insecurity in the relationship functioning as a trigger for this type of behaviors [27,28]. The results observed in the longitudinal study carried out by Cyr and collaborators [29], in a sample of mother-child dyads followed from 1 to 5 years old, went in the same direction: there is an association between coercive parenting and aggression only in children with insecure attachment.

However, and to the best of our knowledge, only one study has investigated the mediation or moderation effects in adolescence, the crucial developmental stage for the implications of the management of aggressiveness. Gallarin and Alonso-Arbiol [30] conducted a study in a sample of late adolescents; their results supported a model of full mediation in which parental control and support would explain maternal and paternal attachment security and where paternal attachment would exert an effect on aggressiveness. The age range analyzed by these researchers, though, was between 16 and 18. Taking into account the need to adapt and regulate parenthood in the first years of adolescence [11,20], a differential effect of parental practices and attachment on aggressiveness may be expected in early adolescence as compared to late adolescence.

### 1.1. Parenting throughout Adolescence

Adolescence is a period in which important changes occur at bio-psycho-social levels with implications for girls’ and boys’ emotional, cognitive, and behavioral development [19,31,32]. In order to better reflect the changes that occur throughout the stage, some authors distinguish between early and late adolescence [11,33]. While early adolescence (between 12–15 years) would be an adaptation phase where boys and girls mostly acquire information and experiences related to the new life stage, late adolescence (between 16 and 18 years old) is a period in which previously acquired information would be used to build and consolidate their own identity [34]. In early adolescence, there are more conflictive episodes, the hierarchy of family roles is more questioned, and there are more discrepancies between parents and offspring [11,35]. This implies that parenting dynamics need to be modified and reorganized—first, from childhood to early adolescence, and second, from early to late adolescence—[36,37]. In that conflictive stage, the most positive affect-related practices—support, closeness, and warmth—decrease from childhood to early adolescence [37], and behavior-related parenting practices—monitoring, control, and discipline—become especially salient, as compared to late adolescence [20].

Several studies account for this evolution across adolescence phases in terms of parental practices. For example, Hoeve and colleagues [38] observed in their meta-analysis that the relationship between parenting practices (i.e., low parental monitoring, rejection, and hostility) and delinquency is stronger in early adolescence than in late adolescence. Other studies have examined antisocial behavior as an outcome, parental practices showing a stronger impact on younger adolescents [39,40,41]. Overall, the results seem to corroborate the higher effect of parenting practices in the first stage of adolescence. Although one may expect such a differential effect for aggressiveness too, hitherto, literature only reveals associations for delinquency and antisocial behavior (i.e., a proxy for direct aggression).

### 1.2. Gender, Parenting, and Aggressiveness

While the mothers’ role in parenting has been extensively examined, the fathers’ role has been more frequently neglected, and therefore, the need to include the paternal figure linked to children’s and adolescents’ well-being is increasingly recognized [42,43]. Fathers’ effect on offspring is thought to be equally important and independent of that of the mother [44]; while the main function of the maternal role seems to be to facilitate love and security, the paternal figure’s role would be to encourage exploration (for a review, see [45]). As for the acceptance dimension of parenting, a gender difference has also been pointed out: paternal acceptance seems to be more closely associated with the decrease of externalizing outcomes—e.g., delinquency and drug use—than maternal acceptance, while the contrary parental effect (i.e., maternal acceptance is more associated than paternal acceptance) is true for internalized outcomes—e.g., self-esteem and self-worth—(for a review, see [46]). This specificity in the role distribution also seems to be maintained throughout adolescence; yet, some distinctions would apply.

As children enter adolescence, interactions with parents gradually start to be based more on conversations, negotiations, and joint decision-making than on unilateral control of behavior [20]. This may provide an explanation as to why aspects such as the handling of closeness, communication, and affection, or conflict resolution can become more prominent. A distance from parents and an increase in the frequency of conflicts characterize the beginning of adolescence [11]. In late adolescence, though, relationships tend to become less conflictive and more intimate, which would explain why conflict moments are experienced with higher intensity [20]. Adolescents increasingly tend to spend more time with mothers and are more likely to share their emotions with them (as compared to fathers), while they turn to fathers for information or material support around specific issues, as these are usually be seen as distant authority figures [19]. This distance could explain why the father figure tends to represent a more coercive role that sets clearer limits (especially in early adolescence), whereas the relationship with the mother is more intense—i.e., closer and more conflictive [20].

Regarding adolescent aggressiveness and the possible differential role of each parental figure, different studies seem to agree in highlighting the paternal function. In the review carried out by Hoeve and colleagues [38], fathers’ behavior of lack of support appeared as more strongly associated with delinquency than mothers’. Likewise, the results of Lansford and colleagues’ [47] longitudinal study indicate that, although maternal and paternal control predict changes in behavior problems during early adolescence, only paternal control accounted for unique variance in adolescent behavior problems.

All these aforementioned studies highlight the differential gender effect of parenting on adolescents’ externalizing (objective) outcomes. A more direct association with aggressiveness has not been commonly addressed. We are aware of one study that specifically examined gender differences in parenting and adolescent aggressiveness; Gallarin and Alonso-Arbiol [30] observed that only paternal (and not maternal) attachment security mediated the relationship between parental practices (acceptance/involvement and coercion/imposition) and aggressiveness in late adolescence. Yet, research in early adolescence has been neglected. Nevertheless, and taking into account that limit-setting and control practices are more needed (and therefore, salient) in early adolescence, we would expect that attachment would not mediate the relationship between those parenting practices and early adolescents’ aggressiveness. Moreover, since the paternal role more commonly represents an authority figure, the effect of paternal parenting on aggressiveness could be higher than of maternal parenting.

### 1.3. Adopted Adolescents and Aggressiveness

Some meta-analytic and systematic reviews have concluded that adopted adolescents show a wide range of difficulties in cognitive, behavioral, emotional, and interpersonal domains [48,49]. It seems that these types of problems put them at a high risk of mental health troubles, especially externalizing-behavioral problems, in comparison to their non-adopted peers (for some reviews, see [50,51]). However, even though adverse experiences before adoption can make adopted adolescents more likely to develop aggression problems, only a few studies have focused on the aggressiveness domain in adopted teens specifically [52]. In any case, in spite of this increased risk, there is also relevant evidence demonstrating that many adoptees exhibited resilience [50,53]. Thus, differences between adopted and non-adopted adolescents could be attributable to individual differences rather than to group differences. In fact, recent adoption research has centered on determining the factors associated with individual differences in adjustment outcomes [54]. In this regard, the adoptive family environment—and particularly, adoptive parents’ behaviors and parent-child relationships—seems to play a critical role [55,56]. Specifically, as it happens in non-adoptive families, there are studies emphasizing the relevance of attachment issues to explain adopted outcomes [57,58]. In addition, as documented in a recent systematic review [54], the effectiveness of some attachment-oriented family interventions in improving adoptee outcomes also supports the importance of a healing family environment.

The same variables that account for normative non-adopted adolescents’ outcomes can also explain adopted teens’ psychological development. In fact, there is some support to the idea of a common pathway to behavioral difficulties for adopted and non-adopted teens [59]; these authors observed that it was the aggregation of risk factors (IQ, attachment, and parenting) in adolescents and in their families, which was significantly related to maladjustment in both adoptees and non-adoptees. In the same vein, as it is the case in normative non-adopted adolescents [26,60], there is some evidence that secure attachment to adoptive parents can mediate the links between parenting styles and adopted children’s [61] and adolescents’ outcomes [62]. Nevertheless, we are only aware of a study that examined the mediation role of attachment between parenting practices and aggressiveness in adolescence [30], and none of them have analyzed it in adopted teens.

### 1.4. The Present Study

Overall, reviewed studies seem to indicate that limit-setting parental practices become more prevalent in early adolescence and that there is a gender differential effect of parenting on variables related to aggressiveness. We believe that clarifying this gender effect that helps to explain aggressiveness in this crucial stage is paramount since parenting affective and behavioral effects may undergo a change process. To the best of our knowledge, a theoretical model in which both points are integrated in early adolescence has not been tested yet. Likewise, such model testing has not been examined with adopted teens either. This study aims to overcome these limitations.

**Figure 1:**
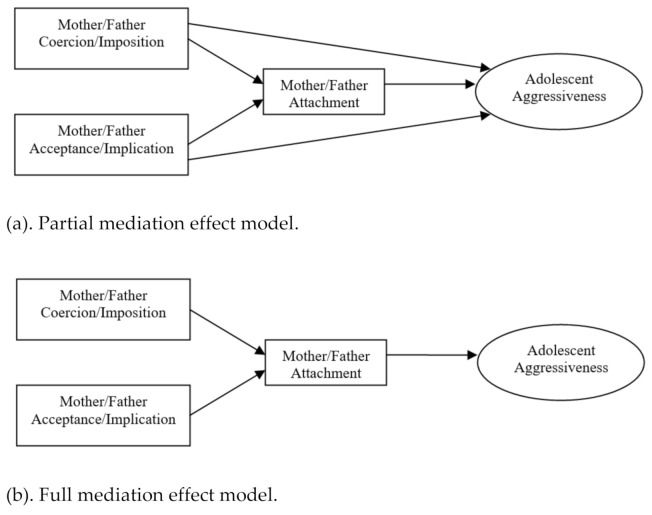
Partial and full mediation models of the relationship between parenting, attachment and aggressiveness.

In an effort to test the model proposed by Gallarin and Alonso-Arbiol [30] (see Figure 1), in which parenting practices and attachment predict adolescents’ aggressiveness, the aims of this study were twofold. First, we sought to test the contribution of parenting practices and attachment security on aggressiveness in the specific stage of early adolescence; and two, to analyze differential effects of mother’s and father’s variables. Moreover, with the added aim of conducting the study from an inclusive perspective that allows testing the model simultaneously for different family realities, it was carried out in two samples of community adolescents: adopted adolescents and non-adopted adolescents. Taking into account previous results in which differences in aggressiveness have not been observed between adopted and non-adopted adolescents [52], it was expected that the model would hold for both adolescent groups. Based on the expected changes to the model for early adolescence, we tested the following hypotheses:

**Hypotheses** **1** **(H1).** *Partial mediation model will fit the data better than the full mediation model for both samples: Parental practices—acceptance/implication and coercion/imposition—will have a direct effect on aggressiveness.*

**Hypotheses** **2** **(H2).** *Paternal practices will be more directly associated with aggressiveness as compared to maternal practices.*

## 2. Materials and Methods

### 2.1. Participants and Procedure

The sample has been drawn from a broader study where different community family types took part—single-parent families, two-parent families, homoparental families, and reconstituted families. Specifically, participants in the present study were 157 Spanish secondary-school students (92 girls and 65 boys) between 12 and 16 years old (mean (M) = 13.87, SD = 1.42). There were two subsamples in the study: (1) Adoptive subsample: 67 adolescents (61.2% girls, age M = 13.84, SD = 1.39) living with their adoptive mother and father and (2) Non-adoptive subsample: 90 adolescents (56.7% girls, age M = 13.89, SD = 1.46) living with their biological mother and father. Taking into account the aims of the study, the existence of a significant relationship with two parents of different sex was used as the criterion for inclusion, leaving single-parent, reconstituted, and homoparental families out of the study (see Table 1 for sociodemographic characteristics of participating adolescents’ families).

Different procedures were developed with each subsample. To recruit adolescents living with birth parents, in the first stage, schools were randomly selected, taking into account their private or public status for the randomization. Principals were contacted for permission granting. Afterward, upon parents’ consent, questionnaires were administered to students in the classroom.

For the adoptive subsample, the collaboration was made with different non-governmental organizations. The associations’ managers were informed about the study. Interested adoptive parents were asked to contact the research team. After consents were obtained, the most suitable dates and places (families’ homes or university lab) for filling in the questionnaires were set up.

Participants were informed about the aims and conditions of the study, and willingness and anonymity were assured. The study had previously received the approval of the authors’ university ethics committee.

### 2.2. Instruments

#### 2.2.1. Parental Socialization

Parental Socialization Scale in Adolescence (Escala de Socialización Parental en la Adolescencia, ESPA-29 [63]) was used to assess perceived paternal and maternal parenting practices. This questionnaire assesses fathers’ and mothers’ socialization practices via two general dimensions: Acceptance/Involvement and Coercion/Imposition, each of which is calculated independently for each parent. Individuals are asked to rate 29 situations (16 positive and 13 negative) on a four-Likert scale of frequency (1 = “never”, 4 = “always”). Acceptance/Involvement is a composite derived from the sum of Communication (“s/he talks to me”) and Affect (“s/he is affectionate with me”), with the subtraction of the scores of Lack of Interest and Indifference (“s/he does not care”) dimensions. Coercion/Imposition derives from the sum of the other three scales: Verbal Coercion (“s/he scolds me”), Physical Coercion (“s/he hits me”), and Deprivation (“s/he deprives me of something”). Father and mother versions yield to independents scores. ESPA-29 has good psychometric properties, including the invariance for sex, age, and country [64]. Cronbach’s alphas of general dimensions for mother and father in this study were all good: 0.92 for mother’s Acceptance/Involvement; 0.94 for mother’s Coercion/Imposition; 0.93 for father’s Acceptance/Involvement; 0.95 for father’s Coercion/Imposition.

#### 2.2.2. Attachment Security

Attachment security was measured using the Inventory of Parent and Peer Attachment (IPPA [65]; in its Spanish version [66]). This self-report assesses attachment security toward mother, father, and peers. In this study, only father and mother versions were used. Each version is made of 16 items to be rated by individuals on a five-Likert scale (from 1 = “never or almost never” to 5 = “always or almost always”). Higher scores are indicative of higher (perceived) secure attachment to the target person/role—mother or father—(e.g., “My mother/father understands me”). Cronbach’s alphas in this study were good: 0.84 for the mother version and 0.88 for the father version.

#### 2.2.3. Aggressiveness

Multifacet Aggressiveness Scale (MAS [30]) was applied to capture aggressiveness. This self-report instrument assesses aggressiveness in an integrative wayproviding independent scores for four subscales: indirect aggression (IA, 12 items), direct aggression (DA, nine items), cognitive dimension of aggressiveness (CD, eight items), and emotional dimension of aggressiveness (ED, 11 items). IA refers to manipulating acts made to damage the social image or relationship network of the target person, or to aggression types that do not need to happen face-to-face (“If I’m angry with someone, I speak about that person with others to make a fool of him/her in front of them”). DA taps forms of aggression in which the victim may identify the perpetrator (“If someone provokes me enough, I can hit him/her”). CD addresses thoughts or desires related to harming someone (“I think that there are people who don’t deserve to be respected”). ED captures the difficulty to manage anger or impulsivity in an adaptive or adjusted way (“I think that I get angry very fast”). MAS is made of 40 items to be rated by the respondent on a five-point Likert answer format, ranging from 1 = ‘Not at all’, to 5 = ‘Very much’. Higher scores would indicate higher levels of aggressiveness. In our study, Cronbach’s alphas were all good: 0.89 for DA, 0.87 for IA, 0.83 for CD, and 0.91 for ED.

### 2.3. Analytic Strategy

We performed Structural Equation Modeling (SEM) analysis using SPSS Statistics (IBM, Madrid) and AMOS 23.0 (IBM, Madrid, Spain) [67]. Partial and full mediation models were analyzed in multi-group structural equation models (using maximum likelihood estimates), allocating non-adoptive and adoptive adolescents into two different groups. The baseline was an unconstrained model in which all parameters were allowed to vary, and subsequent analyses constrained parameters to being invariant in the search for the most parsimonious model that still showed an acceptable fit. Taking into account that the sample size was large and the conventional chi-square statistic is sensitive to sample size [68], several additional indexes were calculated to test the goodness of fit of the models. The relative or normed chi-square is the chi-square fit index divided by its degrees of freedom (χ^2^/df), and values of three or less are seen as pointing to a good fit [69]. Goodness of fit index (GFI), Tucker–Lewis index (TLI), and comparative fit index (CFI) greater than 0.90 were considered to indicate a good fit [70], and values of root-mean-square error approximation (RMSEA) and root-mean-square residual (RMR) lower than 0.05 indicated a good fit [71]. Moreover, in this study, the Akaike Information Criterion (AIC) was used to select the most parsimonious model indicated by the lowest value and lowest decrement between AIC values (Δχ^2^).

## 3. Results

**Figure 2:**
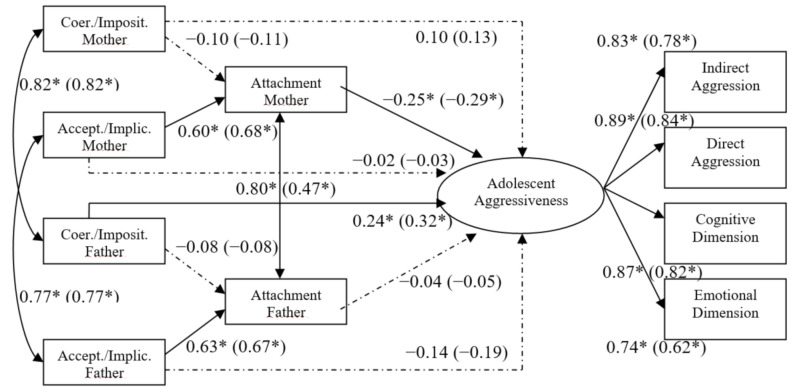
Partial mediation effects model. *Note.* Coer./Imposit. = Coercion/Imposition; Accept./Implic. = Acceptance/Implication. In brackets non-adoptive sample’s regression weights. Dashed arrows indicate statistically non-significant paths. * *p* < 0.05.

As can be seen in Table 2, although both mediation models showed acceptable values in almost all indexes, the partial mediation model had the most favorable fit statistics. Once the full mediation model had been discarded, the lowest AIC in the structural covariances model indicated the most parsimonious option. This model implies invariance for both samples of adolescents. Despite the fact that invariance had been proved, separate values (standardized coefficients) for non-adoptive and adoptive adolescents in the paths of the models are provided in Figure 2, for future possible systematic reviews on adoption research. Moreover, interestingly the association between attachment security toward mother and attachment to father was lower for non-adopted adolescents than for adopted ones. Therefore, H1 was partially supported because only coercion/imposition paths, and not acceptance/implication, show betas of considerable magnitude.

Regarding H2, the results showed a differential effect of paternal and maternal variables in aggressiveness. Whereas each parent’s acceptance/involvement (not coercion/imposition) predicted attachment security to him or her respectively, only father’s coercion/imposition predicted aggressiveness, and only attachment security to mother was a (negative) predictor of adolescent’s aggressiveness. This direct association between paternal coercion and aggressiveness confirms partially Hypothesis 2; for the other paternal practice—acceptance/involvement—was not confirmed, though.

Finally, and taken into account that the model in non-adoptive and adoptive samples did not differ, participants of the two subsamples were grouped together in order to examine the applicability of the model across gender. We conducted a multi-group invariance analysis with two categories (girls and boys). Structural residuals model showed the most favorable fit values (χ2 = 112.64; *p* = 73; CFI = 0.96; TLI = 0.95; RMSEA = 0.06). This model also had the lowest AIC value (AIC = 186.64) and, therefore, the best trade-off between model fit and model complexity, which confirms that the model was invariant for girls and boys.

## 4. Discussion

This study examined the effects of parenting practices and attachment security on aggressiveness, assessing the distinct contribution of mothers and fathers in a sample of adopted and non-adopted early adolescents. The findings here supported the suggestion of a direct effect of paternal coercive behaviors on adolescents’ aggressiveness, whereas the effect of maternal coercion was mediated by attachment security. This model is valid for adopted and non-adopted girls and boys. Extending previous findings on attachment mediation on parenting and aggressiveness [30], our results show the relevance of assessing separately early and late adolescence phases for a better depiction of the different developmental dynamics throughout adolescence. Furthermore, the present study confirms the expected relations between acceptance/involvement and strictness/imposition, as observed in studies in Spain with other indicators (e.g., [72]), extending here the evidence for multidimensional aggressiveness.

Our results support a partial mediation model where acceptance/involvement of both parents predicted attachment security towards them and where father’s coercion/imposition and maternal attachment security predicted adolescent’s aggressiveness. These findings are somewhat different from those found by Gallarin and Alonso-Arbiol [30] in late adolescents, where attachment security fully mediated the relationship between parenting and aggressiveness. In addition, a negative effect of coercion/imposition in attachment security was found in the model for late adolescents examined by Gallarin and Alonso-Arbiol, whereas we did not observe such effect in our model for younger teens. Idiosyncratic features of adolescence stages may explain this difference. In late adolescence, girls and boys have enough cognitive and emotional skills [73] to integrate parents’ both disciplinary and affective practices in the representation of teens’ attachment security [12]. Thus, attachment would mediate between both coercion/imposition and acceptance/implication parenting practices. Unlike late adolescents, early teens do not have such cognitive and emotional maturity to integrate the different facets of the parental practices in their attachment security representation. These younger adolescents would differentiate between coercive and affective interactions with parents, as an instance of concrete thinking [74] or “black and white” reasoning [75]. This idea is reflected in the approach of mentalization framework, whose development is supported by working internal models underlying attachment [76]. Mentalization is understood as a type of imaginative skill to perceive and interpret own and others’ behavior in terms of intentional mental states—(i.e., desires, feelings, needs, and goals [77]. As such, changes are identified in the developmental stage of adolescence. Research has shown that mentalizing capacities improve with age, being better in late than in early adolescence [78,79]. Thus, it is sensible to think that attachment-based mentalizing capacities of older teens allow them a more reflective position to understand and integrate the two dimensions of parental rearing practices that underlie parental mental states. Stemming from here, attachment security would not mediate between coercion/imposition and aggressiveness at this adolescent stage. Yet, future research should test this tentative explanation in a joint sample of early and late adolescents.

The prediction of a more direct association of paternal (vs. maternal) practices in aggressiveness was partially corroborated due to the direct effect of paternal coercion/imposition. Nevertheless, our results also show an interesting non-anticipated mediation of maternal attachment in the relation between acceptance/involvement and aggressiveness that was not observed in the model of Gallarin and Alonso-Arbiol [30] with late adolescents. These results are congruent with previous gender role division found in parenting and attachment. As Koehn and Kerns [25] observed in their meta-analysis, there is a stronger association between teens’ attachment security and responsive parenting in mothers, while fathers seem to play an authority role. Accordingly, in our study, mothers’ affection was more relevant in predicting aggressiveness in early adolescence, whereas the father’s effect was directly associated with coercive behaviors. Therefore, our findings seem to be in line with a traditional gender pattern in the parental functions’ distribution—i.e., mother linked to affection and father associated to authority; younger teens’ lower capacity to integrate both elements reinforces the perception of this role division. In contrast, late adolescents are more capable of integrating the affection-linked and coercion parenting practices, as well as different aspects of attachment experiences in a single overarching attachment organization due to the consolidation of formal operational thinking stage [12] and enhanced mentalizing skills. Moreover, in older teens, the sources of affection diversifies as the relevance of peers as attachment figures increases [41,80]. This joint influence of several attachment figures and of evolving (perceived) gender role division needs to be corroborated in future studies with adolescents of all ages.

As for possible differential influence that mothers’ and fathers’ parenting practices could have on children’s and adolescents’ development, both methodology and the source(s) of information (i.e., informant) could be important factors to take into account. For instance, in a study carried out by John, Halliburton, and Humphrey [81] with mother-child and father-child dyads (under 2 to 4.5 years), different results were found depending on the assessment method. While the qualitative analysis derived from the observation of interactions during the play showed differences between fathers’ and mothers’ reports, in the quantitative analysis carried out as a result of the coding of such behaviors, no differences were found in any dimensions except for one. From the meta-analysis by Hendriks et al. [82], it could be concluded that self-reported parenting was weakly associated with observed parenting. Evidence of associations between self-report and observational measures is reported more frequently in multi-method evaluations of negative rating than in positive rating of parenthood, possibly due to the greater social desirability of positive parenting elements [83]. In addition, certain variables more commonly reported in women, such as distress [84] or depression [85], may also exert some influence on the subjective perception that parents have about their own parenting. Regarding information sources, it seems that, in general, parents’ self-reports could be more favorable than children’s reports on their parents’ behavior [86]. Furthermore, the discrepancy was greater when parents reported more negative parenting or more negative child behavior. The discrepancy between parents and observers on negative child behavior was also predicted by the child’s gender. For boys, parents reported higher levels of negative child behavior than observed but, for girls, parents reported lower levels of negative child behavior than observed [87]. Future studies could clarify these unresolved questions regarding fathers’ and mothers’ differential effect on child-adolescent development.

An additional new insight of our study regards the ecological validity of the present model. Previous findings have acknowledged the need of more studies examining in-depth the specific mechanisms that contribute to the promotion of the development of attachment security in adopted teens [57,88] and of the effect of attachment in aggressiveness [52]. In spite of adopted adolescents’ more difficult previous rearing experiences—e.g., loss, maltreatment, neglect [89]—the present study showed that the contribution of coercion/imposition and acceptance/involvement parenting practices to attachment security and aggressiveness was the same in these teens as in non-adopted counterparts. This result gives additional support to the idea of a common pathway to behavioral difficulties for adopted and non-adopted teens [59].

Despite the aforementioned contributions, the present study was not conducted without limitations. Firstly, the non-randomized design of the study somewhat limited the impetus in the potential generalization of our results. Secondly, although the study posed a heuristic model, its cross-sectional nature impeded an unequivocal conclusion regarding the direction of the relationship among variables in the model. Thirdly, the effect of adolescents’ aggressiveness on parenting practices was not assessed, even though the bidirectional and reciprocal influence in parent-teens relationships has already been noted [24]. Further studies may include longitudinal designs to complement our findings. Finally, the non-significance for some paths may be related to a problem of statistical power, which could be solved in further studies in the future by including larger samples. Furthermore, having bigger samples would allow testing a model with more paths that were not examined here, such as the impact of mother’s acceptance/involvement and strictness/imposition on father’s attachment and vice versa.

Future research may be proposed, stemming from the present study. First, the model could be enhanced by including other types of parental control in the model—i.e., behavioral control and psychological control [37]. Taking into account the differential emotional and behavioral elements of parental control types [90,91], assessment of a wider range of control types, along with attachment, may provide a more accurate view of parents-adolescents dynamics that contribute to the development of adolescent aggressiveness. Moreover, the inclusion of peers as new attachment figures in the model would allow for a better understanding of friends’ specific contribution to aggressiveness as compared to parental attachment. Finally, and bearing in mind the traditional gender pattern observed in our results, future research could examine the effects of parenting and attachment in other family types—particularly homoparental and monoparental families—without such gender role division or in other cultural settings (i.e., not in Spain) where less marked and balanced parental division is the parenting norm. Finally, some potential clinical and psychoeducation applications may be derived from the results of the present study. Specifically, mentalization-based interventions may be implemented to reduce aggressiveness and related problems in adolescents. Focusing on interventions with teenagers, promoting their mentalizing skills is a central theme since mentalization can be a protective factor to prevent the emergence of aggression even in spite of psychopathic traits [92]. In a recent work by Taubner, Gablonski, and Fonagy [93], the authors presented a modification of the Mentalization-Based-Treatment (MBT) for conduct disorder in adolescence. In our view, one of the main strengths of this therapeutic approach is the inclusion and integration of work on attachment and mentalization, both with teenagers and their families. Particularly, enhancing reflective functioning in parents can be a way to promote more positive parenting practices in both mothers and fathers (e.g., [94]). This incipient applied work opens the gate for future research.

## 5. Conclusions

Findings in the present study highlight the importance of examining early and late adolescence phases separately for a better understanding of the parental factors explaining the development of aggressiveness. The following two elements accounts for a traditional gender pattern in the parental role contributing to early teens’ aggressiveness: (a) the direct effect of paternal coercion in adolescents’ aggressiveness, and (b) the attachment-mediated effect of maternal acceptance/implication. In addition, our results support the validity of the tested model for both groups of adolescents (adopted and non-adopted), emphasizing the relevance of parents-teens’ relationships to the understanding of adolescents’ aggressiveness. Professionals might benefit from the results of the present study in designing and implementing intervention and education programs for parents and youth.

## Figures and Tables

**Table 1:** Sociodemographic characteristics in non-adoptive and adoptive families.

	Non-Adoptive Families (*n* = 90) %	Adoptive Families (*n* = 67) %
Family type		
Married	74.2	86.8
Cohabiting	11.9	6.6
Divorced or separated	13.9	6.6
Parental education		
No studies	2.5	-
Primary	15.2	9.4
Secondary	36.2	29.3
University	46.1	61.3
Parents’ employment status		
No employment	15.1	16.1
Employed	83.8	82.3
Pensioner	1.1	1.6
Number of offspring per family		
1	25.6	31.3
2	60.0	49.3
3 or more	14.4	19.4

**Table 2:** Invariance Analysis of Models for Parenting, Attachment and Aggressiveness in non-adoptive and adoptive adolescents.

Model No.	Model Description	χ^2^	*df*	χ^2^*/df*	GFI	TLI	RMSEA	CFI	RMR	AIC	Nested Models	Ʌχ^2^	Ʌ*df*	Sig. Level
Full mediation														
1	Unconstrained	89.16	56	1.59	0.91	0.94	0.06	0.96	0.05	197.16				
2	Measurement weights	89.69	59	1.52	0.91	0.95	0.06	0.97	0.04	191.69	2–1	0.53	3	0.91
3	Structural weights	103.16	65	1.59	0.89	0.94	0.06	0.96	0.06	193.16	3–2	13.47	6	0.04
4	Structural covariances	111.61	73	1.53	0.89	0.95	0.06	0.96	0.06	185.61	4–3	8.45	8	0.39
5	Structural residuals	130.87	77	1.70	0.87	0.93	0.07	0.94	0.06	196.87	5–4	19.26	4	0.01
6	Measurement residuals	132.50	83	1.60	0.87	0.94	0.06	0.95	0.06	186.50	6–5	1.63	6	0.95
Partial mediation														
1	Unconstrained	64.58	48	1.35	0.93	0.97	0.05	0.98	0.03	188.58				
2	Measurement weights	64.90	51	1.27	0.93	0.97	0.04	0.99	0.03	182.90	2–1	0.32	3	0.96
3	Structural weights	78.76	61	1.29	0.92	0.97	0.04	0.98	0.04	176.76	3–2	13.86	10	0.18
*4*	*Structural covariances*	*87.22*	*69*	*1.26*	*0.91*	*0.97*	*0.04*	*0.98*	*0.04*	*169.22*	*4*–*3*	*8.45*	*8*	*0.39*
5	Structural residuals	108.21	73	1.48	0.89	0.95	0.06	0.96	0.05	182.21	5–4	21.00	4	0.01
6	Measurement residuals	110.07	79	1.39	0.89	0.96	0.05	0.97	0.05	172.07	6–5	1.86	6	0.93

*Note.* The model with the best fit appears in italics.

## Data Availability

The data presented in this study are available on request from the corresponding author.
